# Editorial: Peer review vs. open posting

**DOI:** 10.1120/jacmp.v13i5.4090

**Published:** 2012-09-06

Recently, while attending a reunion of my college graduating class, I got into a conversation with a former classmate of mine regarding the subject of peer review of journal manuscripts. My friend questioned the need for peer review, instead advocating a system whereby authors would post manuscripts on a website and readers would post reviews and critiques much as one presently does with websites such as TripAdvisor.

He noted several advantages of such a system. First, manuscripts would be made available for dissemination much more rapidly than they are under peer review. The JACMP presently averages about six months from submission of a manuscript to acceptance, with another three months or so until publication. This time period includes the time it takes for the referees to prepare their reviews of a manuscript, for an author to respond to the reviewers' comments and revise the manuscript in response to the reviewers' comments, and for the referees to review the manuscript a second time. Sometimes a manuscript goes through a third or even fourth round of review, further increasing the time lag from submission to publication. If there were no review, a manuscript could be posted immediately, and the information presented in the manuscript could be shared with the readership community much more rapidly.

The second advantage of such a system is the democratization of the review process. Every reader of a manuscript would become a potential reviewer, allowing for a variety of opinions as to the relative strengths, weaknesses, and utility of a manuscript. Instead of the reviewers being an “elite” class of experts, all medical physicists who read the manuscript would have the opportunity to provide input into the review process.

My friend raised some valid points and, in fact, the idea of open review has been proposed for this, as well as for other, journals. Moreover, when I travel, I often take advantage of TripAdvisor for various travel recommendations. However, medical physics manuscripts are not hotel or restaurant recommendations, and I remain an advocate for peer review for the following reasons:

1. The JACMP is an archival journal, and as an archival journal, is often cited. Consequently, the text of a manuscript needs to be fixed. If it were not fixed, one might reference information in a manuscript only to find that the manuscript has been changed. If an author were to respond to comments posted on a website, and such responses led to a modification of an article, then the journal would no longer be archival, and references to it might no longer be valid. By going through the peer review process, portions of a manuscript that could benefit by change are identified and changed before publication, making it less necessary to modify the text of a manuscript after publication. Modification of a published peer‐reviewed article, if needed to correct an error in a manuscript, is still possible in the form of an Erratum.
2. Most readers of journal articles have neither the time nor the inclination nor the responsibility to provide detailed reviews of manuscripts. Peer reviews of journal submissions are often quite detailed and demonstrate a significant contribution of time and effort of the reviewers who have been selected specifically for their expertise in the topic of the manuscript, and who have made a commitment to providing a thorough review of the manuscript.
3. Authors have typically spent a great deal of time and effort in the specific discipline of their manuscript and may fail to recognize the limitations of expertise of their readership. An unbiased, uninvolved third party can often identify points in a manuscript that need clarification or explanation. The need for recognizing the expertise of the readership is especially important for a journal such as the JACMP, whose readership constitutes a large cross section of clinical medical physicists, as opposed to a research journal for which the readership of a particular manuscript may be limited to individuals pursuing research in the same specific discipline as the author.
4. Medical physicists are not necessarily the best writers in the English language, even those for whom English is their native language. All JACMP manuscripts (including this editorial), as well as manuscripts in other peer‐reviewed journals, are copyedited for style and grammar, which leads to improvements in the language upon publication.
5. Academic medical physicists need to publish in peer‐reviewed journals for consideration of promotion and tenure. Posting a manuscript on a website just does not count as much toward promotion as publishing a manuscript in a peer‐reviewed journal.
6. We acknowledge the criticism that peer review leads to a longer time between work and publication. The need for rapid dissemination of information is much more important for a research journal than it is for a clinical journal. Research can move very quickly, and the information presented in a research journal is often used to develop more information; consequently, rapid dissemination of research information is highly desirable. Information presented in a clinical journal is used to improve the quality of clinical practice; rapid dissemination of such information is not as significant an issue.
7. Several listservers and bulletin boards already exist in which medical physicists are able to post ideas and exchange them with their colleagues. These postings are brief and may not be the result of a careful study. It is up to the reader of these postings to determine the validity of the posting. Moreover, these postings occasionally include impolitic or discourteous responses, which are inappropriate for a medium of scientific communication.
8. Finally, to address the criticism that peer review tends to be elitist, and does not allow the readership of a manuscript to comment and discuss the manuscript — that statement just is not true for the JACMP. Comments on any manuscript can be posted on our website. Just go to the manuscript, either in PDF or HTML format, and click on the “Post a Comment” link on the right side of the page. We medical physicists are not in the habit of posting comments, but here at the JACMP we have provided the option and you, the readership, are encouraged to do so. Consequently, if you have reached this point in the Editorial, please post a comment on it; agree or disagree — your opinions are welcomed.

In our conversation, my friend raised some valid points, but I am quite comfortable with the concept of peer review, and hope that the JACMP will continue to be a peer‐reviewed journal.

George Starkschall, PhD

Editor‐in‐Chief

September 6, 2012

